# Genetic Ancestry and Genome-Wide Association Study Combined with Functional Enrichment Analyses Reveal Candidate Genes for Body Conformation Traits in Hexi Cattle

**DOI:** 10.3390/ani16142216

**Published:** 2026-07-16

**Authors:** Xinlu Wang, Bin Ma, Zhicheng Wang, Yicheng Liu, Xiaoming Ma, Min Chu, Yongfu La, Xian Guo, Ping Yan, Lei Wang, Chunnian Liang

**Affiliations:** 1 Key Laboratory of Animal Genetics and Breeding on Tibetan Plateau, Ministry of Agriculture and Rural Affairs, Lanzhou 730050, China; 2 Key Laboratory of Yak Breeding of Gansu Province, Lanzhou Institute of Husbandry and Pharmaceutical Sciences, Chinese Academy of Agricultural Sciences, Lanzhou 730050, China; 3 Zhangye Livestock Breeding and Improvement Workstation, Zhangye 734000, China

**Keywords:** Hexi cattle, genome-wide association study, body conformation traits, population structure, candidate genes

## Abstract

Hexi cattle are a local cattle population endemic to the Hexi Corridor in Gansu Province, China, and exhibit strong adaptability to the region’s arid continental environment. However, comprehensive genomic investigations of this population are still lacking. In the present study, we integrated population genomic analyses with a genome-wide association study (GWAS) to dissect the genetic architecture of Hexi cattle. Whole-genome resequencing data were generated for 264 Hexi cattle, and public genomic datasets from six representative cattle breeds were obtained from the NCBI database for comparative analysis. Multiple analytical approaches—including principal component analysis (PCA), linkage disequilibrium (LD) decay analysis, neighbor-joining (NJ) phylogenetic tree construction, and ADMIXTURE analysis—were adopted to evaluate population structure and evolutionary relationships. A mixed linear model was then used to identify significant SNPs associated with five major body conformation traits in six-month-old cattle: body weight (BW), withers height (WH), hip height (HH), heart girth (HG), and abdominal girth (AG). Our results confirm the admixed nature of Hexi cattle, whose genome is derived primarily from Simmental cattle and secondarily from Mongolian cattle. A total of 69 trait-associated significant SNPs were identified and functionally annotated. Specifically, *TBC1D31*, *DERL1* and *MCPH1* were linked to BW; *FSCN3* and *PLBD1* to WH; *MCPH1* to HH; *NPAS3*, *TBC1D31*, *DERL1* and *MCPH1* to HG; and *SOX5*, *NTAQ1*, *FAM83A*, *TBC1D31*, *DERL1*, *CDH11*, *CPLX4* and *ADAM18* to AG. This study deepens our understanding of the genetic basis of growth traits in Hexi cattle and offers valuable molecular resources for future selective breeding, genetic improvement, and long-term conservation of this indigenous cattle population.

## Simple Summary

Hexi cattle are an indigenous population originating from the Hexi Corridor of China, renowned for their robust growth performance and excellent adaptability to arid climates. However, their genetic background and the genes governing body size traits remain poorly characterized. In this study, we conducted a genomic analysis of 264 six-month-old Hexi calves and compared them with six other cattle breeds. The results indicated that Hexi cattle represent a distinct genetic group, with ancestry primarily derived from Simmental and Mongolian cattle. Through a genome-wide association study (GWAS), we identified 69 molecular markers associated with five key growth traits: body weight, withers height, hip height, heart girth, and abdominal girth. Several candidate genes, including *SOX5*, *TBC1D31*, *DERL1*, *MCPH1* and *CDH11*, were found to be correlated with these phenotypic traits. These findings elucidate the genetic mechanisms underlying growth characteristics in Hexi cattle and provide valuable molecular markers for selective breeding. Breeders can apply these markers to screen individuals with superior growth potential, enhance beef productivity, and facilitate the sustainable utilization and conservation of this precious local cattle breed.

## 1. Introduction

Local livestock breeds constitute irreplaceable genetic resources that have undergone long-term evolution under combined natural and artificial selection pressures. To enhance production performance while maintaining inherent adaptability to local habitats, systematic crossbreeding between indigenous cattle breeds and introduced commercial breeds has been widely applied in global livestock breeding programs—an approach fully validated by previous research [1]. Hexi cattle originated in Northwest China through planned multi-generational crossing between maternal East Asian Mongolian cattle and paternal European Simmental cattle. Through successive phenotypic and genomic selection, they have acquired a distinctive admixed genome. Thanks to such crossbred ancestry and sustained artificial selection, Hexi cattle harbor a typical admixed genetic architecture, which renders them an ideal model for deciphering genomic and phenotypic alterations shaped by directional artificial selection. Their unique genetic composition stems from marked differences in adaptive capacity and growth performance between their two progenitor breeds: Mongolian cattle are renowned for outstanding cold tolerance and disease resistance, whereas Simmental cattle excel in growth rate and superior production performance.

Mongolian cattle represent a typical East Asian taurine subspecies, characterized by prominent cold tolerance, strong disease resistance, and excellent environmental adaptability [2]. In comparison, Simmental cattle are well-known dual-purpose breeds for both beef and milk production, featuring fast growth rate, large body size, and high lean meat percentage [3]. Additionally, minor genetic introgressions from other European beef cattle breeds, such as Limousin and Charolais, further enrich the overall genetic diversity of Hexi cattle [4]. Nevertheless, the accurate ancestral composition, fine-scale admixture patterns, and detailed population substructure of Hexi cattle have not been fully clarified. Such unsolved scientific issues limit in-depth germplasm evaluation of this indigenous population and further restrict the application of marker-assisted selection (MAS) and other modern molecular breeding technologies for its genetic improvement [5].

Whole-genome sequencing (WGS) can effectively fill this research gap. Unlike conventional low-resolution methods, WGS provides base-pair resolution across the genome. This resolution is essential for reconstructing complex admixture histories and detecting subtle population substructures in crossbred cattle [6]. As a fundamental tool for high-resolution genomic analysis, WGS allows precise estimation of global ancestral proportions, identification of local ancestry tracts, and calculation of admixture time. Its reliability has been validated in numerous admixed cattle populations [7,8]. Therefore, WGS acts as an indispensable and reliable strategy to dissect the population genetic structure of Hexi cattle. This technology can identify millions of genome-wide SNPs, supporting high-resolution analysis of population structure and demographic history, and its application value in bovine genomic research has been widely acknowledged [9]. Accumulating genomic studies have verified the power of WGS to untangle cattle admixture backgrounds. For instance, WGS results revealed that Guanling cattle have a mixed genetic background containing 50% East Asian indicine ancestry and 35% East Asian taurine ancestry, accompanied by high genetic diversity and low inbreeding coefficients [10]. Similarly, in Xinjiang Brown cattle, obvious ancestry differences were observed across different feeding modes: grazing cattle had an admixed genome consisting of 37.22% Brown Swiss ancestry and 62.78% Kazakh cattle ancestry, whereas feedlot-fed cattle were dominated by Brown Swiss ancestry [11]. Another WGS study reported that Kazakh White-Headed cattle harbor an admixed genome with approximately 45% Hereford, 30% Altai, and 25% Kalmyk ancestry [12]. Additionally, WGS-based evidence showed that improved Qinchuan cattle and Zaosheng cattle share similar genomic origins; both breeds carry higher proportions of European taurine ancestry than the original Qinchuan cattle, and such genetic variation is closely associated with their enhanced body size [13]. Collectively, these previous studies demonstrate that WGS is the gold-standard approach for clarifying fine-scale admixture patterns and tracing ancestral origins in crossbred cattle. Genome-wide association studies (GWASs) have been widely adopted to uncover functional candidate genes responsible for livestock economic traits [5]. For example, a large-scale GWAS based on 39,135 Holstein dairy cows successfully detected multiple genetic loci closely linked to cattle body conformation traits [14]. Similarly, a sequence-based GWAS of 14,762 Belgian Blue beef cattle identified *LCORL-NCAPG* and *CCND2* as strong candidate genes for body conformation traits [15]. In addition, a GWAS conducted on indigenous Gudali cattle and their Simmental crossbred offspring (Simgud) screened numerous significant trait-related SNPs and annotated core functional genes participating in skeletal development, muscle proliferation, and energy metabolism [16]. Moreover, another GWAS involving 183 Simmental cows pinpointed genetic variants associated with body conformation performance, tail morphological characteristics, and metabolic inflammation pathways [17]. Collectively, these research outcomes provide solid theoretical and empirical support for the application of marker-assisted selection (MAS) and genomic selection (GS) in modern cattle breeding systems.

At present, the genetic architecture governing growth and body conformation traits of Hexi cattle is still poorly understood. Given the above research deficiencies, the present study aimed to systematically clarify the population genetic structure and ancestral composition of Hexi cattle by comparing this breed with six representative cattle breeds and to further screen functional candidate genes related to critical body conformation traits via GWAS. The results of this study will offer reliable genomic references for rational genetic improvement and standardized marker-assisted selection of this precious indigenous beef cattle breed.

## 2. Materials and Methods

### 2.1. Animals, Body Conformation Measurements, and Blood Collection

A total of 264 clinically healthy Hexi cattle (125 males and 139 females) were obtained from three core breeding farms located in Zhangye City, Gansu Province. All animals were raised under standardized nutritional and management conditions. Five key body conformation traits, including body weight (BW), withers height (WH), hip height (HH), heart girth (HG), and abdominal girth (AG), were systematically measured. All body weight and conformation measurements were collected in the morning from fasting animals by experienced staff at the local livestock research station, following the Technical Specifications for Beef Cattle Production Performance Measurement (GB/T 43838-2024) [18]. For blood sampling, approximately 5 mL of venous blood was collected from the jugular vein of each Hexi animal using sterile vacuum tubes containing EDTA-K_2_ as an anticoagulant. Immediately after collection, all blood tubes were gently inverted 5–8 times to achieve complete blending of blood and anticoagulant. Subsequently, all qualified blood samples were stored at −20 °C until subsequent genomic DNA extraction.

### 2.2. DNA Extraction and Sequencing

Genomic DNA was isolated from whole blood samples using the TIANamp Genomic DNA Kit (Tiangen Biotech Co., Ltd., Beijing, China) according to the manufacturer’s protocol. The concentration and purity of extracted DNA were determined via a NanoDrop 2000 spectrophotometer (Thermo Fisher Scientific, Waltham, MA, USA). In addition, DNA integrity and overall quality were further evaluated by 1% (*w*/*v*) agarose gel electrophoresis and a Qubit^®^ 3.0 fluorometer (Thermo Fisher Scientific), respectively. Finally, high-quality DNA samples that met sequencing standards were delivered to Smart Biotech (Qingdao, China) for whole-genome resequencing.

### 2.3. Data Quality Control and Population Genetic Structure Analysis

In this study, whole-genome resequencing was performed on 264 Hexi cattle. In addition, publicly available genomic sequencing data of six reference cattle breeds, including Angus, Holstein, Limousin, Mongolian cattle, Simmental and Charolais, were retrieved from the NCBI database under the BioProject accession PRJEB42783. Raw sequencing reads were mapped to the bovine reference genome ARS-UCD1.2 (bosTau9, GCF_002263795.1) using BWA-MEM (v0.7.17). The resulting alignment files were further processed and converted to coordinate-sorted BAM files using SAMtools (v1.9) [19]. The average sequencing depth was 20x ross all sequenced individuals. SNPs were called using GATK (v4.2.6.1) with default parameters [20], and subsequent functional annotation of qualified SNPs was conducted via SnpEff (v5.0). PLINK (v1.90) was used for genotype quality control [21]. During quality filtering, only autosomal SNPs satisfying the following criteria were retained: genotyping call rate > 90% (--geno 0.1), minor allele frequency (MAF > 0.01), and Hardy–Weinberg equilibrium (HWE > 1 × 10^−5^). Additionally, SNPs located on sex chromosomes and mitochondrial DNA, as well as variants with unknown genomic positions, were removed. Ultimately, a total of 22,613,679 high-quality SNPs were obtained for subsequent population genomics and GWAS analyses.

To clarify the population genetic structure of Hexi cattle and their phylogenetic relationships with six reference cattle breeds (Angus, Holstein, Limousin, Mongolian cattle, Charolais, and Simmental), principal component analysis (PCA) was performed on the merged high-quality SNP dataset using PLINK (v1.90). The first five principal components (PCs) were extracted, and the top two PCs explaining the maximum genome-wide genetic variance were selected for subsequent visualization. PCA scatter plot was generated using ggplot2 (v4.5.1) to reveal population clustering patterns and pairwise genetic differentiation among all enrolled cattle breeds. Genome-wide linkage disequilibrium (LD) decay was further evaluated using PopLDdecay (v3.42), in which the squared correlation coefficient (r^2^) for each pair of SNPs was calculated within 1 Mb sliding windows. LD decay curves were plotted based on the mean r^2^ values corresponding to different genomic physical distances. To eliminate sample size bias affecting LD comparison results, 30 individuals were randomly chosen from the Hexi cattle population to match the sample size of most reference breeds, and the detailed sample size for each breed was presented as follows: Hexi cattle (*n* = 30), Angus (*n* = 40), Holstein (*n* = 30), Limousin (*n* = 30), Mongolian cattle (*n* = 16), Charolais (*n* = 30), and Simmental (*n* = 30). Pairwise individual Euclidean genetic distances were calculated based on the top two PCs, and a neighbor-joining (NJ) phylogenetic tree was constructed and visualized using ggtree (v3.12.0) coupled with ggplot2 (v4.5.1). In addition, model-based population admixture analysis was performed using ADMIXTURE v1.3.0 by setting ancestral population numbers ranging from K = 2 to K = 7, and the optimal K value was determined according to the minimum cross-validation (CV) error; finally, individual and population ancestry proportions extracted from Q-matrices were visualized via integrated tidyverse packages (v2.0.0) in R (v4.3.1) [22].

### 2.4. Genome-Wide Association Study and Candidate Gene Annotation

A mixed linear model (MLM) was adopted to perform genome-wide association analysis for five growth-related body conformation traits measured in 6-month-old Hexi cattle. To eliminate confounding factors that might interfere with association results, breeding farm and animal sex were included as fixed effects. In addition, the first five genome-wide SNP-based principal components (PCs) were added as fixed covariates to correct population stratification. Furthermore, a genomic kinship matrix was fitted as a random effect to adjust for individual cryptic relatedness and polygenic background effects across the study population. The formula of the applied mixed linear model was as follows:

y = Xα + Zβ + Wμ + e(1)

where y is the vector of phenotypic traits; X is the design matrix for fixed effects, and α is the vector of estimated fixed effects (including farm, sex, and the first five PCs); Z is the SNP genotype matrix, and β is the vector of SNP effects to be estimated; W is the random effect matrix, the genomic kinship matrix was fitted as the random effect, and μ stands for the vector of polygenic background effects structured by the genomic kinship matrix; e is the vector of random errors.

To control the false positive risk due to multiple testing, this study set the genome-wide significance threshold at *P* = 1 × 10^−6^, which is consistent with previous studies [23]. To evaluate the influence of population stratification on the GWAS results, we calculated the genomic inflation factor (λ) and generated quantile–quantile (Q-Q) plots for the five growth traits using R (v4.3.1).

### 2.5. Functional Enrichment Analysis

The biological functions of candidate genes identified via genome-wide association analysis were explored using Gene Ontology (GO) functional annotation and Kyoto Encyclopedia of Genes and Genomes (KEGG) pathway enrichment analysis. GO analysis categorized these candidate genes into three functional dimensions, namely, cellular component (CC), biological process (BP), and molecular function (MF), while KEGG analysis was performed to screen significantly enriched biological signaling pathways. All enrichment analyses were carried out with the clusterProfiler package (v4.0). in R (v4.3.1), using the cattle (Bos taurus) annotation database org.Bt.eg.db (v3.21.0) as the background reference. The Benjamini–Hochberg method was used for multiple testing correction, and an adjusted *p* < 0.05 was set as the significance threshold. For intuitive visualization of gene functional profiles, GO enrichment results were presented with a Sankey diagram, and all KEGG enrichment outcomes were summarized in a formal table.

## 3. Results

### 3.1. Phenotypic Data Statistics of Each Body Size Trait

Table 1 shows the descriptive statistics for body conformation traits of 6-month-old Hexi cattle. All measured traits were significantly higher in males than in females, with the most notable differences observed for BW and AG. BW showed the largest coefficient of variation (17–19%), while the coefficients of variation for WH, HH, HG, and AG ranged from 4.4% to 8.7%. HH exceeded WH in all animals. Furthermore, AG was significantly larger than HG in male individuals.

### 3.2. Population Structure and Kinship Analysis

**Figure 1:**
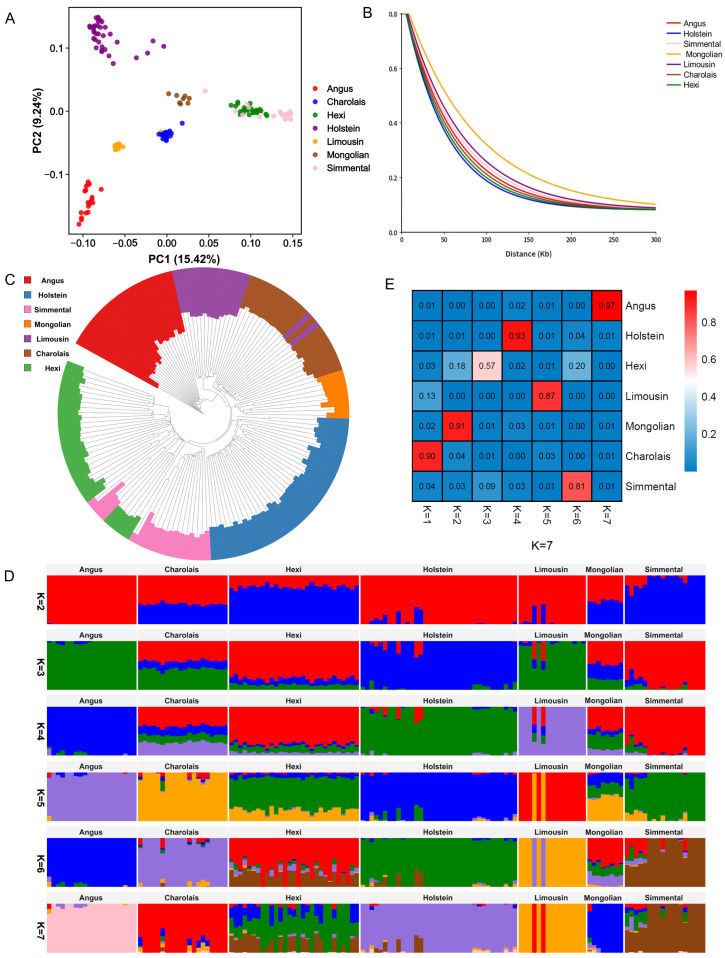
Population genetic structure and evolutionary relationships among cattle breeds. (**A**) PCA plot; (**B**) LD decay plot; (**C**) phylogenetic tree; (**D**) ADMIXTURE ancestry component profiles of seven cattle breeds at K = 2 to K = 7; Each bar indicates one individual, with colors representing distinct ancestral genetic li neages; Breed-specific genetic backgrounds and interbreed admixture signals are displayed; (**E**) heatmaps of average ancestral components across populations.

PCA based on the first two PCs revealed that Hexi cattle formed a genetically distinct cluster, clearly separated from the other six breeds (Figure 1A). LD decay analysis using a 1 Mb sliding window revealed slower LD attenuation and longer LD blocks in Hexi cattle compared with Angus, Holstein, Limousin, Mongolian, Simmental, and Charolais (Figure 1B). The NJ phylogenetic tree built from pairwise genetic distances further validated the PCA results, showing that Hexi cattle formed a single clade most closely related to Simmental cattle (Figure 1C). Admixture analysis was performed for K values ranging from 2 to 7 (Figure 1D). Cross-validation error determined K = 7 as the optimal number of ancestral populations, which could fully distinguish all reference breeds. Under this model, Simmental ancestry dominated the genome of Hexi cattle at 57%, followed by 18% Mongolian ancestry (Figure 1E).

### 3.3. Genome-Wide Association Study of Different Body Size Traits

**Figure 2:**
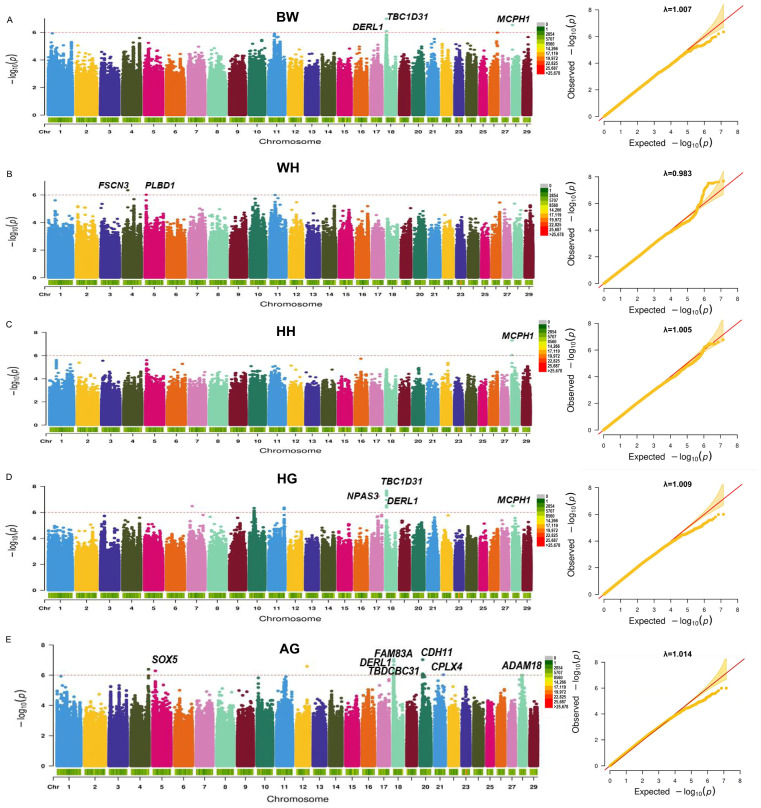
Manhattan and QQ plots of body conformation traits in Hexi cattle. (**A**): Body weight (BW); (**B**): withers height (WH); (**C**): hip height (HH); (**D**): heart girth (HG); (**E**): abdominal girth (AG).

The genomic inflation factors (λ) for BW, WH, HH, HG, and AG were 1.007, 0.983, 1.005, 1.009, and 1.014, respectively, indicating minimal population stratification and robust GWAS results. A total of 69 significant SNPs were identified for five body conformation traits, of which 59 received successful gene annotations (Table S1), and the other 10 remained unannotated. In detail, five SNPs were significantly associated with BW and annotated to *TBC1D31*, *DERL1* and *MCPH1* (Figure 2A); three SNPs were linked to WH and corresponded to *FSCN3* and *PLBD1* (Figure 2B); two SNPs were associated with HH and annotated to *MCPH1* (Figure 2C); twenty SNPs were correlated with HG and mapped to *NPAS3*, *TBC1D31*, *DERL1* and *MCPH1* (Figure 2D); and thirty-nine SNPs were significantly related to AG and annotated to *SOX5*, *NTAQ1*, *FAM83A*, *TBC1D31*, *DERL1*, *CDH11*, *CPLX4* and *ADAM18* (Figure 2E). Three pleiotropic candidate genes were found to affect multiple body conformation traits: *DERL1* was associated with BW, HG and AG; *MCPH1* influenced BW, HH and HG; and *TBC1D31* contributed to variations in BW, HG and AG.

### 3.4. Functional Enrichment Analysis

**Figure 3:**
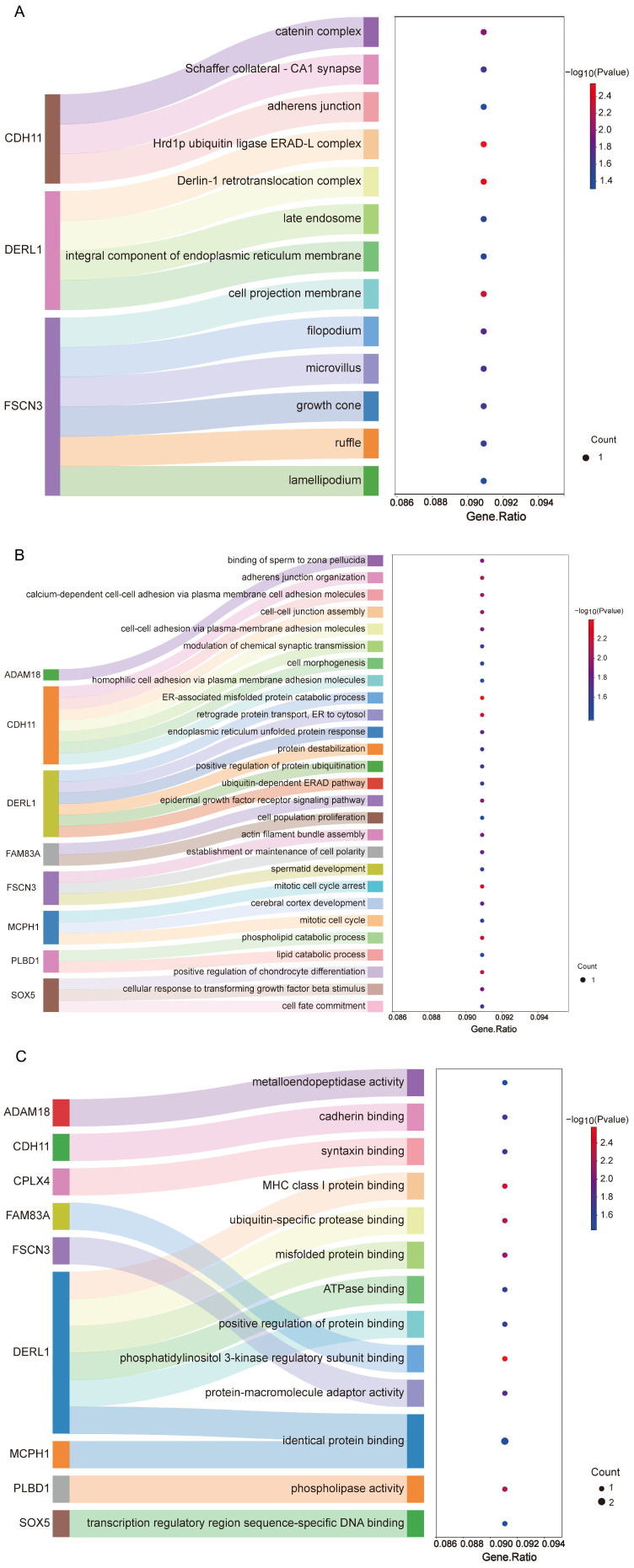
Functional enrichment results of the annotated candidate genes from the GWAS in Hexi cattle. (**A**): GO enrichment plot for Cellular Component; (**B**): GO enrichment plot for Biological Process; (**C**): GO enrichment plot for Molecular Function.

GO enrichment analysis further revealed the functional profiles of these candidate genes. In the cellular component category, corresponding proteins were mainly distributed in the growth cone, Derlin-1 retrotranslocation complex, adherens junctions, and Hrd1p ubiquitin ligase ERAD-L complex (Figure 3A). For biological processes, genes were enriched in lipid catabolic process, mitotic cell cycle progression, calcium-dependent cell–cell adhesion via plasma membrane cell adhesion molecules, cell fate commitment, and binding of sperm to zona pellucida (Figure 3B). In terms of molecular function, enriched terms included transcription regulatory region sequence-specific DNA binding, positive regulation of protein binding, phosphatidylinositol 3-kinase regulatory subunit binding, syntaxin binding, and metalloendopeptidase activity (Figure 3C). Subsequent KEGG pathway enrichment analysis revealed two significantly enriched pathways: *DERL1* was enriched in the ALS pathway, and *CPLX4* was enriched in the synaptic vesicle cycle pathway (Table S2).

### 3.5. Genotyping of Single-Nucleotide Polymorphism Loci

**Figure 4:**
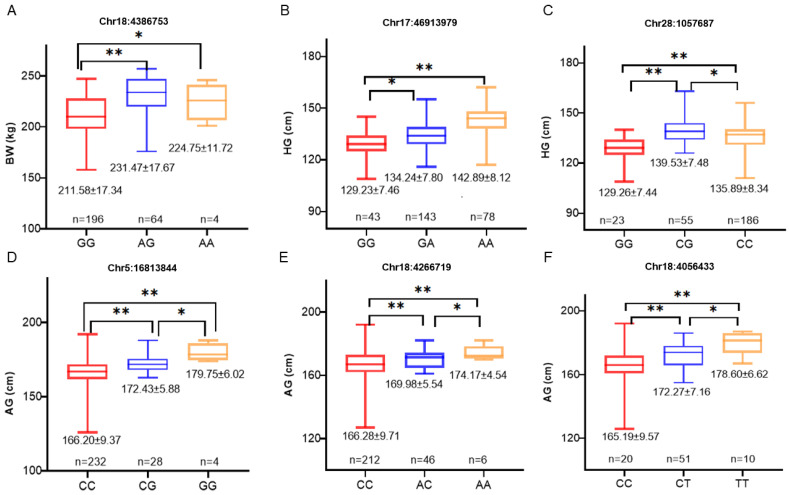
Genotyping results for key SNP loci in Hexi cattle. (**A**): Chr18:4386753; (**B**): Chr17:46913979; (**C**): Chr28:1057687; (**D**): Chr5:16813844; (**E**): Chr18:4266719; (**F**): Chr18:4056433. significant relationships among the three genotypes. * indicates that 0.01 ≤ *p* < 0.05, ** indicates that *p* < 0.01.

Six significantly associated SNP loci (Chr18:4386753, Chr17:46913979, Chr28:1057687, Chr5:16813844, Chr18:4266719, and Chr18:4056433) were used for visualization. Each locus was annotated to a putative candidate gene: *DERL1*, *NPAS3*, *MCPH1*, *SOX5*, *FAM83A*, and *NTAQ1*, respectively. Genotyping of 264 Hexi cattle showed highly significant differences (*p* < 0.01) in the relevant phenotypic traits between individuals homozygous for the wild-type allele and those homozygous for the mutant allele at each locus (Figure 4).

## 4. Discussion

Hexi cattle are a native dual-purpose population distributed across the Hexi Corridor of Gansu Province, featuring favorable growth potential and superior adaptability to extreme local environments. Our results showed that male Hexi cattle exhibited significantly superior performance in all body conformation traits at 6 months of age relative to females, with body weight and abdominal girth showing the most prominent sexual dimorphism. Such sex-dependent differences in early growth patterns have been widely reported in mainstream beef cattle breeds, including Angus, Charolais, and Hereford [24]. Similar growth differences between sexes are also common in small ruminants, with male lambs growing faster and exhibiting heavier body weight than female counterparts [25,26]. In terms of body conformation, all individuals measured in this study exhibited higher hip height than withers height, forming a typical “low forequarter and high hindquarter” body shape, which is regarded as an ideal conformation for beef and dual-purpose cattle production. In addition, male Hexi cattle had a larger AG than HG; although female cattle showed a similar trend, the gap between the two measurements was much smaller. Abdominal girth can directly reflect abdominal cavity capacity, which is closely linked to rumen development, feed intake, and nutrient digestion efficiency in cattle [27]. Specifically, male calves experience rapid rumen development at six months of age, which expands abdominal cavity volume, increases abdominal girth, and further accelerates body weight gain. By comparison, female calves preferentially allocate energy to basic physiological maintenance rather than rapid somatic growth at the same developmental stage, which accounts for the non-significant difference between their abdominal girth and heart girth [28].

In this study, we comprehensively investigated the population genetic structure of Hexi cattle using three complementary analytical methods: PCA, LD decay analysis, and genetic ancestry analysis. PCA results revealed that Hexi cattle form an independent genetic cluster clearly separated from other beef cattle breeds. Notably, Hexi cattle exhibited the closest genetic relationship with Simmental cattle, consistent with historical crossbreeding events and ongoing gene flow between the two populations [29]. Phylogenetic analysis further supported the above results. Although Hexi cattle formed a stable monophyletic branch, incomplete lineage sorting was observed between Hexi cattle and Simmental cattle, indicating recent and ongoing genetic admixture. Such genetic admixture is common in indigenous livestock breeds with long-term human-mediated gene introgression, which is driven by intensive artificial selection, population demographic changes, and inbreeding [30]. LD decay analysis revealed slow LD attenuation and longer genome-wide LD blocks in Hexi cattle, which provides evidence of strong and long-term artificial selection pressure on its genome. This genomic feature has also been reported in many domestic livestock breeds under intensive directional selection [31,32]. Ancestry composition analysis revealed that Simmental cattle serve as a superior paternal breed for growth trait improvement, and persistent artificial selection has profoundly remodeled the genomic composition of Hexi cattle [33]. The predominant Simmental genetic ancestry also indicates that Simmental cattle have been widely used as sires to improve the growth performance of local cattle in historical crossbreeding programs. In addition, the stable Mongolian cattle ancestral component (approximately 18%) identified in Hexi cattle is consistent with historical documents stating that Mongolian cattle were the original indigenous cattle population in the Hexi Corridor. This conserved genetic component contributes to the strong environmental adaptability of Hexi cattle, such as drought tolerance and efficient utilization of low-quality forage, allowing this breed to adapt to the arid ecological conditions of Northwest China [34].

Early growth traits, including BW, WH, HH, HG, and AG, are crucial economic traits for beef cattle breeding [35]. GWAS serves as an efficient tool to screen candidate genes responsible for these complex polygenic traits and explore their molecular regulatory mechanisms [36]. In this study, we conducted a whole-genome resequencing-based GWAS on 6-month-old Hexi cattle and detected several pleiotropic genomic loci significantly associated with growth traits in cattle. Among these candidate genes, *SOX5* was identified as a key gene affecting abdominal girth. Copy number variations in the *SOX5* gene are significantly associated with withers height and heart girth in yaks [37]. Similarly, *TBC1D31* shows a significant association with body weight in Hexi cattle, which is consistent with earlier research on Hanwoo cattle that verified a tight link between this gene and body size characteristics [38]. The transcription factor gene *NPAS3* also exhibits evolutionarily conserved functions in growth regulation across species: a prior GWAS in Large White pigs revealed that *NPAS3* was significantly associated with body weight and backfat thickness [39], consistent with our observation that this gene influences heart girth, an integrated body conformation trait shaped by thoracic muscle growth and subcutaneous fat accumulation. Furthermore, *CDH11* was previously documented to modulate body weight and skeletal development in Mexican beef cattle [40], whereas it was only significantly related to abdominal girth rather than body weight in the Hexi cattle population evaluated herein. This interpopulation discrepancy suggests population-specific genetic effects of *CDH11*, and we hypothesize that this gene may indirectly alter abdominal girth by regulating the development of the thoracolumbar skeleton in Hexi cattle.

This study identified several candidate genes with previously uncharacterized roles in body size regulation, which likely contribute to the growth of Hexi cattle through distinct and biologically coherent pathways. *FAM83A* has been reported to promote adipogenesis and fat deposition by interacting with casein kinase 1α and increasing mitochondrial outer membrane permeability, which could be linked to abdominal girth expansion [41]. As a key component of the endoplasmic reticulum-associated degradation (ERAD) pathway, *DERL1* clears misfolded proteins and orchestrates lipid metabolism, cell cycle turnover, and skeletal muscle homeostasis; notably, it accelerates myofiber proliferation and differentiation, thereby facilitating body weight gain and whole-body growth of this local cattle breed [42]. As a vital guardian of mitotic stability, *MCPH1* ensures orderly proliferation and differentiation of myoblasts and osteoblasts, supporting muscle mass accretion and longitudinal bone growth [43]. Consistently, GO enrichment results confirmed that *MCPH1* is significantly enriched in cell proliferation, mitotic cell cycle, and cell–cell adhesion processes, all of which are indispensable for muscle and bone formation. In addition, *ADAM18* shapes skeletal development by regulating bone matrix degradation, osteoblast migration, and mineralization, and remodels the extracellular matrix to boost myofiber growth and fusion during muscle development [44]. In our study, *ADAM18* showed strong genome-wide association with abdominal girth, a complex phenotype collectively shaped by skeletal configuration and adipose deposition. Collectively, these candidate genes regulate growth traits predominantly via cell cycle regulation, cell adhesion, and extracellular matrix remodeling cascades. However, these bioinformatic inferences remain predictive, and follow-up in vitro cell assays and in vivo functional validation are necessary to confirm their exact biological functions in Hexi cattle. 

## 5. Conclusions

In summary, this study conducted a systematic genomic analysis of Hexi cattle. Hexi cattle carry predominant Simmental lineage, accompanied by a consistent Mongolian cattle genetic component, and this mixed genetic background contributes to their strong environmental adaptability. A total of 69 growth-related SNPs and eight candidate genes, namely, *SOX5*, *TBC1D31*, *NPAS3*, *CDH11*, *FAM83A*, *DERL1*, *MCPH1*, and *ADAM18*, were identified via GWAS. This study clarifies the population genetic structure of Hexi cattle and identified functional molecular markers. The results provide fundamental data for molecular breeding and genetic improvement of this indigenous cattle breed.

## Figures and Tables

**Table 1:** Phenotypic values of body conformation traits in Hexi cattle.

Items	Sex (*n*)	Mean ± SD	Coefficient of Variation (CV)	Min	Max
BW (kg)	Male (125)	226.65 ± 38.70 ^a^	17.1%	168	246
	Female (139)	208.48 ± 39.30 ^b^	18.9%	159	225
WH (cm)	Male (125)	105.36 ± 5.36	4.1%	102	117
	Female (139)	104.11 ± 5.31	5.1%	97	109
HH (cm)	Male (125)	112.30 ± 5.86 ^B^	5.2%	107	124
	Female (139)	110.86 ± 4.88 ^B^	4.4%	101	117
HG (cm)	Male (125)	137.35 ± 9.31 ^A^	5.8%	128	155
	Female (139)	135.03 ± 9.15 ^A^	6.8%	125	146
AG (cm)	Male (125)	171.41 ± 11.61 ^a^	6.7%	158	187
	Female (139)	164.97 ± 14.39 ^b^	8.7%	142	179

Note: BW = body weight; WH = withers height; HH = hip height; HG = heart girth; AG = abdominal girth. ^a,b^ For each trait, values bearing different superscripts indicate significant differences between males and females at *p* < 0.05. ^A,B^ Within the same sex, values bearing different superscripts indicate significant differences between HH and HG at *p* < 0.05.

## Data Availability

The data presented in this study are available on request from the corresponding authors.

## Supplementary Materials

The following supporting information can be downloaded at: https://www.mdpi.com/article/10.3390/ani16142216/s1, Table S1: Significant SNPs and their corresponding annotated genes; Table S2: KEGG pathway enrichment.

## References

[1] Groeneveld L.F., Lenstra J.A., Eding H., Toro M.A., Scherf B., Pilling D., Negrini R., Finlay E.K., Jianlin H., Groeneveld E. (2010). Genetic diversity in farm animals—A review. Anim. Genet..

[2] Su R., Zhou H., Yang W., Moqir S., Ritu X., Liu L., Shi Y., Dong A., Bayier M., Letu Y. (2024). Near telomere-to-telomere genome assembly of Mongolian cattle: Implications for population genetic variation and beef quality. GigaScience.

[3] Mansur M., Diansyah A.M., Rahmat R., Amrullah M.F., Alfian A.M., Adam A.A.S., Nurlatifah A. (2025). Reproductive disorders in Simmental cattle: Enhancing fertility through a hormonal protocol. Open Vet. J..

[4] Wang Y., Li J., Zhang S., Zhang Q., Zhang Y. (2018). Challenges and opportunities for genetic improvement of cattle production in China. J. Anim. Sci..

[5] Zhang Y., Cai W., Zhang Q., Li Q., Wang Y., Peng R., Yin H., Hu X., Wang Z., Zhu B. (2025). Integrated analyses of genomic and transcriptomic data reveal candidate variants associated with carcass traits in Huaxi cattle. J. Integr. Agric..

[6] Kalbfleisch T., Petersen J.L., Tait R.G., Qiu J., Basnayake V., Hackett P.H., Heaton M.P. (2020). Using triallelic SNPs for determining parentage in North American yak (Bos grunniens) and estimating cattle (Bos taurus) introgression. F1000Research.

[7] Wang X., Nursyifa C., Aninta S.G., Garcia-Erill G., Bertola L.D., Khan A., Kuja J., Hanghøj K., Meisner J., Bøggild T. (2025). The genetic diversity of Indonesian cattle has been shaped by multiple introductions and adaptive introgression. Nat. Commun..

[8] McHugo G.P., Ward J.A., Ng’ang’a S.I., Frantz L.A.F., Salter-Townshend M., Hill E.W., O’Gorman G.M., Meade K.G., Hall T.J., MacHugh D.E. (2025). Genome-wide local ancestry and the functional consequences of admixture in African and European cattle populations. Heredity.

[9] Lu Y., Li M., Gao Z., Ma H., Chong Y., Hong J., Wu J., Wu D., Xi D., Deng W. (2025). Advances in whole genome sequencing: Methods, tools, and applications in population genomics. Int. J. Mol. Sci..

[10] Su Z., Xian K., Bian C., Li F., Qi X., Lei C., Xia X. (2026). Genetic admixture and adaptive signatures of Guanling cattle revealed by whole-genome sequence. BMC Genom..

[11] Wang X., Ma Z., Gao L., Yuan L., Ye Z., Cui F., Guo X., Liu W., Yan X. (2023). Genome-wide survey reveals the genetic background of Xinjiang Brown cattle in China. Front. Genet..

[12] Khamzina A.K., Igoshin A.V., Muslimova Z.U., Turgumbekov A.A., Khussainov D.M., Yudin N.S., Ussenbekov Y.S., Larkin D.M. (2025). Resequencing composite Kazakh Whiteheaded cattle: Insights into ancestral breed contributions, selection signatures, and candidate genetic variants. Animals.

[13] Yu H., Zhang K., Cheng G., Mei C., Wang H., Zan L. (2024). Genome-wide analysis reveals genomic diversity and signatures of selection in Qinchuan beef cattle. BMC Genom..

[14] Sousa Junior L.P.B., Pinto L.F.B., Cruz V.A.R., Oliveira Junior G.A., Oliveira H.R., Chud T.S., Pedrosa V.B., Miglior F., Schenkel F.S., Brito L.F. (2024). Genome-wide association and functional genomic analyses for body conformation traits in North American Holstein cattle. Front. Genet..

[15] Gualdrón Duarte J.L., Yuan C., Gori A.S., Moreira G.C.M., Takeda H., Coppieters W., Charlier C., Georges M., Druet T. (2023). Sequenced-based GWAS for linear classification traits in Belgian Blue beef cattle reveals new coding variants in genes regulating body size in mammals. Genet. Sel. Evol..

[16] Matenchi Y.P., Bastanlar E.K., Hegarty M. (2025). Genome-wide association analysis revealed novel candidate genes for body measurement traits in indigenous Gudali and crossbred Simgud in Cameroon. BMC Genom..

[17] Wang J., Shen N., Zhao K., Liao J., Jiang G., Xiao J., Jia X., Sun W., Lai S. (2025). Revealing study and breeding implications for production traits and tail characteristics in Simmental cattle by GWAS. Front. Genet..

[18] (2024). Technical Specification for Measuring the Production Performance of Beef Cattle.

[19] Okonechnikov K., Conesa A., García-Alcalde F. (2016). Qualimap 2: Advanced multi-sample quality control for high-throughput sequencing data. Bioinformatics.

[20] Lin Y.L., Chang P.C., Hsu C., Hung M.Z., Chien Y.H., Hwu W.L., Lai F., Lee N.C. (2022). Comparison of GATK and DeepVariant by trio sequencing. Sci. Rep..

[21] Price A.L., Patterson N.J., Plenge R.M., Weinblatt M.E., Shadick N.A., Reich D. (2006). Principal components analysis corrects for stratification in genome-wide association studies. Nat. Genet..

[22] Alexander D.H., Novembre J., Lange K. (2009). Fast model-based estimation of ancestry in unrelated individuals. Genome Res..

[23] He X.Y., Wei Y.S., Kuang J.C., Li Z.F., Yu Z.X., Wang X.Y., Di R., Zhu C.Y., Chu M.X. (2026). Genome-wide association study identifies candidate genes regulating multi-stage body size traits in Longling yellow goats. Animal.

[24] Doyle J.L., Purfield D.C., Moore T., Carthy T.R., Walsh S.W., Veerkamp R.F., Evans R.D., Berry D.P. (2021). Identification of genomic regions that exhibit sexual dimorphism for size and muscularity in cattle. J. Anim. Sci..

[25] Ghafouri-Kesbi F., Baneh H. (2018). Genetic aspects of sexual size dimorphism in a synthesized breed of sheep. Meta Gene.

[26] Mandal A., Baneh H., Rout P.K., Notter D.R. (2022). Genetic analysis of sexual dimorphism in growth of Jamunapari goats of India. J. Anim. Breed. Genet..

[27] Khan M.A., Weary D.M., von Keyserlingk M.A. (2011). Invited review: Effects of milk ration on solid feed intake, weaning, and performance in dairy heifers. J. Dairy Sci..

[28] Pokhrel B., Jiang H. (2024). Postnatal growth and development of the rumen: Integrating physiological and molecular insights. Biology.

[29] Wu X., Pei J., Xiong L., Ge Q., Bao P., Liang C., Yan P., Guo X. (2025). Genome-wide scan for selection signatures reveals novel insights into the adaptive capacity characteristics in three Chinese cattle breeds. BMC Genom..

[30] Senczuk G., Mastrangelo S., Ciani E., Battaglini L., Cendron F., Ciampolini R., Crepaldi P., Mantovani R., Bongioni G., Pagnacco G. (2020). The genetic heritage of Alpine local cattle breeds using genomic SNP data. Genet. Sel. Evol..

[31] Bordbar F., Jensen J., Wadood A.A., Yao Z. (2024). Linkage disequilibrium decay in selected cattle breeds. Animals.

[32] Cai J., Yang L., Gao Y., Liu G.E., Da Y., Ma L. (2025). Selection signature analysis of whole-genome sequences to identify genome differences between selected and unselected Holstein cattle. Animals.

[33] Poutougnigni Matenchi Y., Hegarty M. (2025). Genomic diversity of Cameroonian Gudali and Gudali-cross cattle. Sci. Rep..

[34] Xu L., Zhou K., Huang X., Chen H., Dong H., Chen Q. (2024). Whole-genome resequencing provides insights into the diversity and adaptation to desert environment in Xinjiang Mongolian cattle. BMC Genom..

[35] Han M., Wang X., Du H., Cao Y., Zhao Z., Niu S., Bao X., Rong Y., Ao X., Guo F. (2025). Genome-wide association study identifies candidate genes affecting body conformation traits of Zhongwei goat. BMC Genom..

[36] Dehghan A. (2018). Genome-wide association studies. Methods Mol. Biol..

[37] Zhang Z., Chu M., Bao Q., Bao P., Guo X., Liang C., Yan P. (2022). Two different copy number variations of the *SOX5* and *SOX8* genes in yak and their association with growth traits. Animals.

[38] Srikanth K., Lee S.H., Chung K.Y., Park J.E., Jang G.W., Park M.R., Kim N.Y., Kim T.H., Chai H.H., Park W.C. (2020). A gene-set enrichment and protein-protein interaction network-based GWAS with regulatory SNPs identifies candidate genes and pathways associated with carcass traits in Hanwoo cattle. Genes.

[39] Yang W., Wu J., Yu J., Zheng X., Kang H., Wang Z., Zhang S., Zhou L., Liu J. (2021). A genome-wide association study reveals additive and dominance effects on growth and fatness traits in large white pigs. Anim. Genet..

[40] Jahuey-Martínez F.J., Parra-Bracamonte G.M., Sifuentes-Rincón A.M., Martínez-González J.C., Gondro C., García-Pérez C.A., López-Bustamante L.A. (2016). Genomewide association analysis of growth traits in Charolais beef cattle. J. Anim. Sci..

[41] Huang K., Jia Z., Li H., Peng Y., Chen X., Luo N., Song T., Wang Y., Shi X., Kuang S. (2022). Proto-oncogene FAM83A contributes to casein kinase 1-mediated mitochondrial maintenance and white adipocyte differentiation. J. Biol. Chem..

[42] Wang T., Wang D., Kuang G., Gong X., Zhang L., Wan J., Li K. (2024). Derlin-1 promotes diet-induced non-alcoholic fatty liver disease via increasing RIPK3-mediated necroptosis. Free Radical. Biol. Med..

[43] Gruber R., Zhou Z., Sukchev M., Joerss T., Frappart P.O., Wang Z.Q. (2011). MCPH1 regulates the neuroprogenitor division mode by coupling the centrosomal cycle with mitotic entry through the Chk1-Cdc25 pathway. Nat. Cell Biol..

[44] Edwards D.R., Handsley M.M., Pennington C.J. (2008). The ADAM metalloproteinases. Mol. Asp. Med..

