# Supplementary material for: Genetic Ancestry and Genome-Wide Association Study Combined with Functional Enrichment Analyses Reveal Candidate Genes for Body Conformation Traits in Hexi Cattle
Source: Animals (Basel). 2026 Jul 16;16(14):2216. doi: 10.3390/ani16142216 (PMC13405829; doi:10.3390/ani16142216)
Supplement: Supplementary file 1 [file animals-16-02216-s001.zip › animals-4360761-supplementary.pdf]

Table S1: Significant SNPs and their corresponding annotated genes

Items	position	Annotation gene	Items	position	Annotation gene
BW	Chr18: 4376479	*TBC1D31*	WH	Chr4:30820194	*FSCN3*
	Chr18: 4386753	*DERL1*		Chr5:7045531; Chr5:7045545	*PLBD1*
	Chr28:1057687	*MCPH1*			
HH	Chr5:1057546; Chr5:1057687	*MCPH1*		Chr5:16813844	*SOX5*
				Chr18: 4266719	*FAM83A*
	Chr17:4371246; Chr17:4376479	*NPAS3*		Chr18:4377354; Chr18:4376479; Chr18:4377354	*TBC1D31*
HG	Chr18:4378679; Chr18:4379354; Chr18:4383458; Chr18:4383654; Chr18:4386753; Chr18:4388335; Chr18:4395261; Chr18:4395964; Chr18:4396993; Chr18:4401172; Chr18:4406530	*DERL1*	AG	Chr18:4378679; Chr18:4379354; Chr18:4383458; Chr18:4383654; Chr18:4386753; Chr18:4388335; Chr18:4395261; Chr18:4395964; Chr18:4396993; Chr18:4401172; Chr18:4406530	*DERL1*
	Chr28:1057687	*MCPH1*		Chr21:57328177	*CPLX4*
				Chr28: 19236454	*ADAM18*
AG	Chr20:16684797; Chr20:16851067; Chr20:1685873; Chr20:16868107; Chr20:16874936; Chr20:1688622; Chr20:16889132; Chr20:16898352; Chr20:1690146; Chr20:16913050	*CDH11*		Chr20:16916336; Chr20:16920179; Chr20:16921809; Chr20:16927100; Chr20:16935579; Chr20:16940597; Chr20:16943781; Chr20:16948409; Chr20:17106981	*CDH11*

Note: Body weight (BW); Withers height (WH); Hip height (HH); Heart Girth (HG); Abdominal girth (AG).

Table S2: KEGG pathway enrichment

gene	KEGG Pathway
DERL1	Amyotrophic lateral sclerosis (ALS)
CPLX4	Synaptic vesicle cycle
